# Early brainstem ^[18F]^THK5351 uptake is linked to cortical hyperexcitability in healthy aging

**DOI:** 10.1172/jci.insight.142514

**Published:** 2021-01-25

**Authors:** Maxime Van Egroo, Daphne Chylinski, Justinas Narbutas, Gabriel Besson, Vincenzo Muto, Christina Schmidt, Davide Marzoli, Paolo Cardone, Nora Vandeleene, Martin Grignard, André Luxen, Eric Salmon, Christian Lambert, Christine Bastin, Fabienne Collette, Christophe Phillips, Pierre Maquet, Mohamed Ali Bahri, Evelyne Balteau, Gilles Vandewalle

**Affiliations:** 1 GIGA-Cyclotron Research Centre-In Vivo Imaging and; 2 Psychology and Cognitive Neuroscience Research Unit, University of Liège (ULiège), Liège, Belgium.; 3 Department of Neurology, University Hospital of Liège, Liège, Belgium.; 4 Wellcome Centre for Human Neuroimaging, University College London Institute of Neurology, London, United Kingdom.; 5 GIGA-In Silico Medicine, ULiège, Liège, Belgium.

**Keywords:** Aging, Neuroscience, Alzheimer’s disease, Neuroimaging

## Abstract

**BACKGROUND:**

Neuronal hyperexcitability characterizes the early stages of Alzheimer’s disease (AD). In animals, early misfolded tau and amyloid-β (Aβ) protein accumulation — both central to AD neuropathology — promote cortical excitability and neuronal network dysfunction. In healthy humans, misfolded tau and Aβ aggregates are first detected, respectively, in the brainstem and frontomedial and temporobasal cortices, decades prior to the onset of AD cognitive symptoms. Whether cortical excitability is related to early brainstem tau — and its associated neuroinflammation — and cortical Aβ aggregations remains unknown.

**METHODS:**

We probed frontal cortex excitability, using transcranial magnetic stimulation combined with electroencephalography, in a sample of 64 healthy late-middle–aged individuals (50–69 years; 45 women and 19 men). We assessed whole-brain ^[18F]^THK5351 PET uptake as a proxy measure of tau/neuroinflammation, and we assessed whole-brain Aβ burden with ^[18F]^Flutemetamol or ^[18F]^Florbetapir radiotracers.

**RESULTS:**

We found that higher ^[18F]^THK5351 uptake in a brainstem monoaminergic compartment was associated with increased cortical excitability (*r* = 0.29, *P* = 0.02). By contrast, ^[18F]^THK5351 PET signal in the hippocampal formation, although strongly correlated with brainstem signal in whole-brain voxel-based quantification analyses (*P* value corrected for family-wise error [*P_FWE-corrected_*] < 0.001), was not significantly associated with cortical excitability (*r* = 0.14, *P* = 0.25). Importantly, no significant association was found between early Aβ cortical deposits and cortical excitability (*r* = –0.20, *P* = 0.11).

**CONCLUSION:**

These findings reveal potential brain substrates for increased cortical excitability in preclinical AD and may constitute functional in vivo correlates of early brainstem tau accumulation and neuroinflammation in humans.

**TRIAL REGISTRATION:**

EudraCT 2016-001436-35.

**FUNDING:**

F.R.S.-FNRS Belgium, Wallonie-Bruxelles International, ULiège, Fondation Simone et Pierre Clerdent, European Regional Development Fund.

## Introduction

Alzheimer’s disease (AD) is characterized by a pathogenesis that spreads over decades prior to the onset of cognitive symptoms (1). Tau neurofibrillary tangles (NFT) and amyloid-β (Aβ) senile plaques play a central role in this process (2). Postmortem studies showed that the accumulation of misfolded hyperphosphorylated tau protein, which eventually assembles into NFT, appears prior to any detectable Aβ lesion (3). By the age of 30, a vast majority of the population presents hyperphosphorylated tau aggregates in the brainstem locus coeruleus (LC), sometimes concomitantly to other brainstem nuclei such as the dorsal raphe (3–5). Tau aggregates and NFT then spread to the entorhinal and hippocampal regions, before following its stereotypical outward progression pattern in the cortex, with about half of the postmortem samples showing some NFT by the sixth decade of life (3). By contrast, Aβ senile plaques are not found before the fifth decade of life, while about 20% of the population present Aβ senile plaques between 50 and 60 years (6). Aβ senile plaques also follow a stereotyped progression pattern, but it is distinct from tau NFT expansion. They are first found in the frontomedial and temporobasal areas, before reaching the rest of the neocortex, allocortical brain regions (e.g., entorhinal cortex and hippocampus), and nuclei from the basal forebrain; they ultimately encompass brainstem nuclei and the cerebellum (6, 7).

Growing evidence supports the idea that AD pathogenesis affects neuronal function well before producing neuronal loss and brain atrophy. Increase in neuronal excitability, which reflects neuron responsiveness and response selectivity, has been reported to temporarily take place before AD symptom onset (8, 9). Motor cortex hyperexcitability was reported in prodromal AD (mild cognitive impairment; MCI) (10, 11), while cortical excitability decreases markedly in more advanced AD (12). Moreover, acetylcholinesterase inhibitor therapy was suggested to restore normal cortical excitability in AD patients (13). This temporary increase in cortical excitability has been proposed to represent a compensatory mechanism to face the early brain Aβ or tau deposits (9). Monitoring cortical excitability could therefore enhance early/presymptomatic risk assessment and diagnosis, and it could help monitoring disease progression.

The release of both tau and Aβ in the extracellular space are regulated by neuronal activity (14, 15). Elevated interstitial and cerebrospinal (CSF) fluid tau and Aβ levels have been observed during periods of extended neuronal activity such as sleep deprivation (16, 17). In AD animal models, tau pretangles and their surrounding soluble hyperphosphorylated form — tau NFTs — Aβ oligomers, and Aβ senile plaques are known to have a deleterious impact on neuronal function (8, 18). In addition, tau presence is associated with inflammatory reaction through increased microglial and astrocytic activation (19, 20), which further contribute to synaptic dysfunction (21–23). However, several studies reported a direct relationship between early tau protein accumulation and increased neuronal excitability in rodents and drosophila models in distant areas (24–26). Likewise, transgenic AD rodent models overexpressing mutant human amyloid precursor protein (APP) revealed that Aβ pathology promotes hyperexcitation and hyperactivity of hippocampus and neocortex neurons (27).

Regional quantification of tau pretangles and soluble hyperphosphorylated forms, as well as Aβ oligomers, remain impossible in vivo in humans, so their direct impact on brain function is unknown. In vivo PET imaging of Aβ senile plaques (Aβ-PET) has been reliably available in humans for almost 2 decades, so their correlates in cognitively normal individuals and patients are relatively well established (28). In contrast, tau NFT in vivo imaging has only been possible over the past 5 years (29). In addition, most first- and second-generation tau PET markers have different degrees of off-binding, including to monoamine oxidase-B (MAO-B), which prevents from isolating the specific consequence of tau NFT from, for instance, neuroinflammatory processes (30).

**Figure 1:**
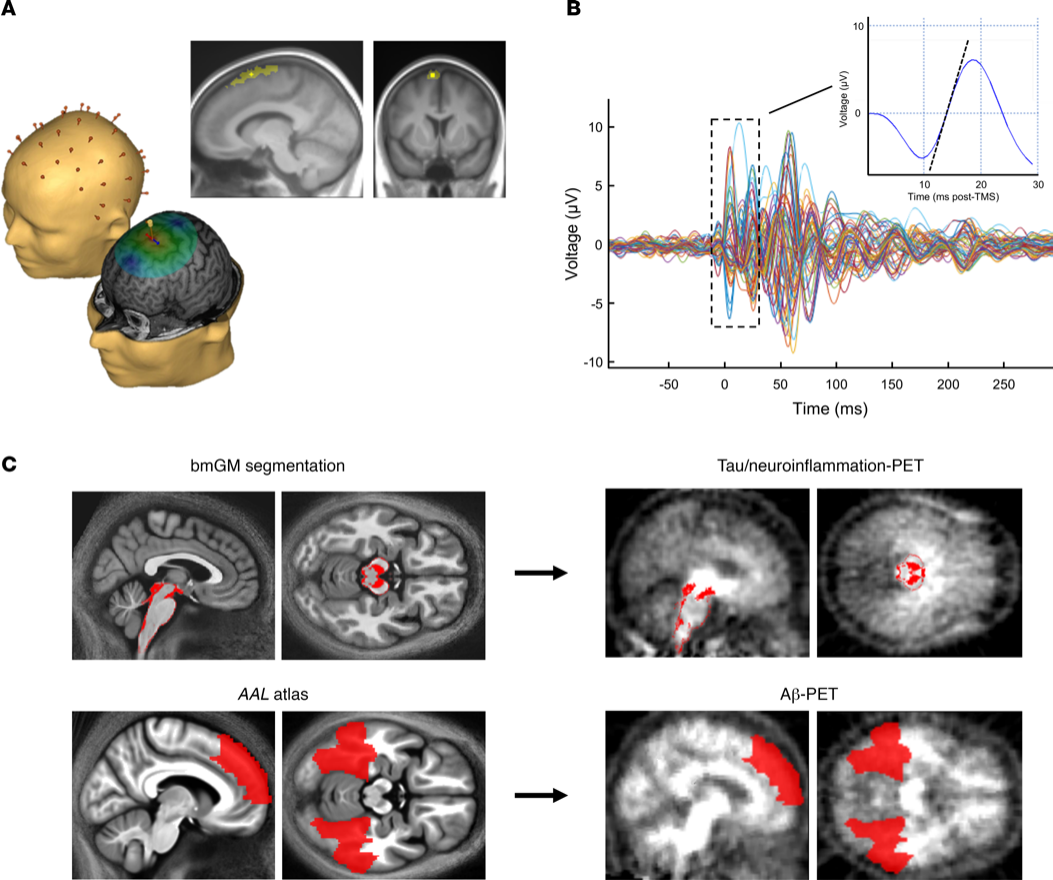
Cortical excitability assessment and PET value extraction in early deposition sites. (**A**) Cortical excitability over the frontal cortex was assessed using neuronavigation-based TMS coupled to EEG. TMS-EEG target area was located in the superior frontal gyrus. Darker and brighter yellow areas represent the range of stimulation targets across participants projected onto the averaged normalized T1 structural volume and the median TMS-EEG stimulation hotspot over the sample, respectively. (**B**) Butterfly plot of TMS-evoked EEG response over the 60 electrodes (–100 ms before TMS to 300 ms after TMS; average of approximately 250 trials). Cortical excitability was computed as the slope (μV/ms, dotted line on inset) of the first component of the TEP response at the electrode closest to the stimulation hotspot. (**C**) Automatic brainstem segmentation methods were used to extract ^[18F]^THK5351 SUVR in the brainstem monoaminergic gray matter (bmGM; top row). Aβ burden (^[18F]^Flutemetamol or ^[18F]^Florbetapir centiloid units) was extracted in the earliest aggregation sites (7) using bilateral medial superior frontal, inferior temporal, and fusiform regions (bottom row).

Here, our main goal was to assess whether, among late-middle–aged cognitively normal individuals (50–69 years), cortical excitability was associated with early tau NFT and neuroinflammation burden and with early Aβ senile plaques. We directly measured cortical excitability using electroencephalogram (EEG) recordings of brain responses to transcranial magnetic stimulation (TMS), which mimics the active brain processing of external stimulation. We assessed whole-brain ^[18F]^THK5351 PET uptake as a proxy of tau NFT/neuroinflammation burden, as well as whole-brain Aβ burden, using ^[18F]^Flutemetamol and ^[18F]^Florbetapir PET imaging. Based on quantitative MRI data, we then extracted ^[18F]^THK5351 and Aβ radiotracer uptake values within their respective first sites of accumulation (i.e., in a compartment of the brainstem including monoaminergic neurons for ^[18F]^THK5351 uptake and in the medial prefrontal cortex and inferior temporal lobe for Aβ-PET; Figure 1 and Table 1). We hypothesized that both brainstem ^[18F]^THK5351 and neocortical Aβ-PET uptake values would be associated with increased cortical excitability.

## Results

**Figure 2:**
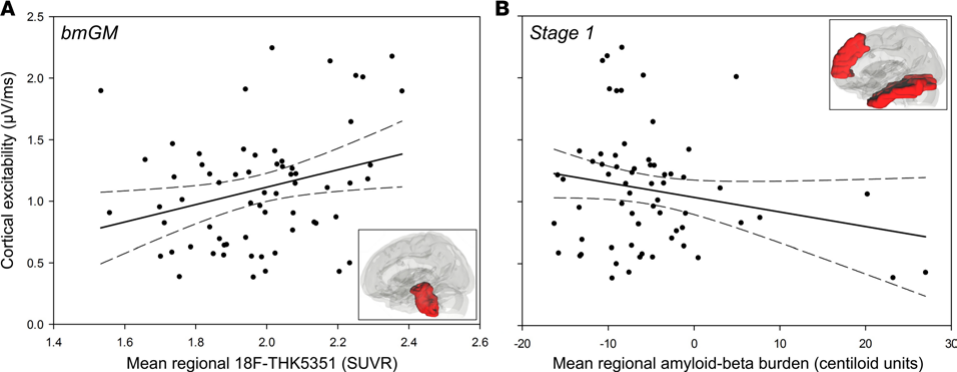
Associations between cortical excitability and early ^[18F]^THK5351 PET signal, as well as Aβ burden. (**A**) Significant and positive association between cortical excitability values and mean ^[18F]^THK5351 standardized uptake value ratio (SUVR) in the brainstem monoaminergic gray matter (bmGM; Pearson’s correlation: *r* = 0.29, *P* = 0.02; GLMM: F_1,57_ = 4.76, *P* = 0.03, R²_β*_ = 0.08). (**B**) No significant association between cortical excitability and mean ^[18F]^Flutemetamol/^[18F]^Florbetapir (centiloid units) in an ROI covering the earliest Aβ aggregation sites (Pearson’s correlation: *r* = –0.20; *P* = 0.11; GLMM: F_1,57_ = 1.41, *P* = 0.24). Simple regressions are displayed, and full GLMM outputs are reported in Table 2. Dotted lines represent 95% CI for these simple regressions. Inset images illustrate the ROI used to extract PET values in each analysis.

Our analysis first focused on the link between cortical excitability, as assessed with TMS-EEG over the frontal cortex (TMS-evoked EEG potential; TEP), and ^[18F]^THK5351 PET signal in the brainstem monoaminergic gray matter (bmGM). We found a significant and positive association between TEP slope and bmGM mean ^[18F]^THK5351 standardized uptake value ratio (SUVR), both in a simple correlation and after adjusting for TMS-EEG stimulation parameters and demographic variables (Pearson’s correlation: *r* = 0.29, *P* = 0.02; generalized linear mixed model [GLMM]: F_1,57_ = 4.76, *P* = 0.03, *R*²**_β*_** = 0.08; Figure 2A and Table 2).

To address the specificity of the highlighted relationship, we then investigated the link between cortical excitability and average Aβ centiloid values in stage 1 regions of interest (ROIs) — i.e., in the earliest sites of senile plaques aggregation. We found no significant relationship between TEP slope and stage 1 ROI Aβ burden (Pearson’s correlation: *r* = –0.20, *P* = 0.11; GLMM: F_1,57_ = 1.41, *P* = 0.24; Figure 2B and Table 2). Interestingly, including both bmGM ^[18F]^THK5351 SUVR and stage 1 Aβ burden together in the same statistical model (i.e., taking into account the variance they respectively explain) still revealed a significant main effect of bmGM ^[18F]^THK5351 values (GLMM: F_1,56_ = 5.78, *P* = 0.02, *R*²**_β*_** = 0.09) but not of stage 1 Aβ burden (Table 2).

**Figure 3:**
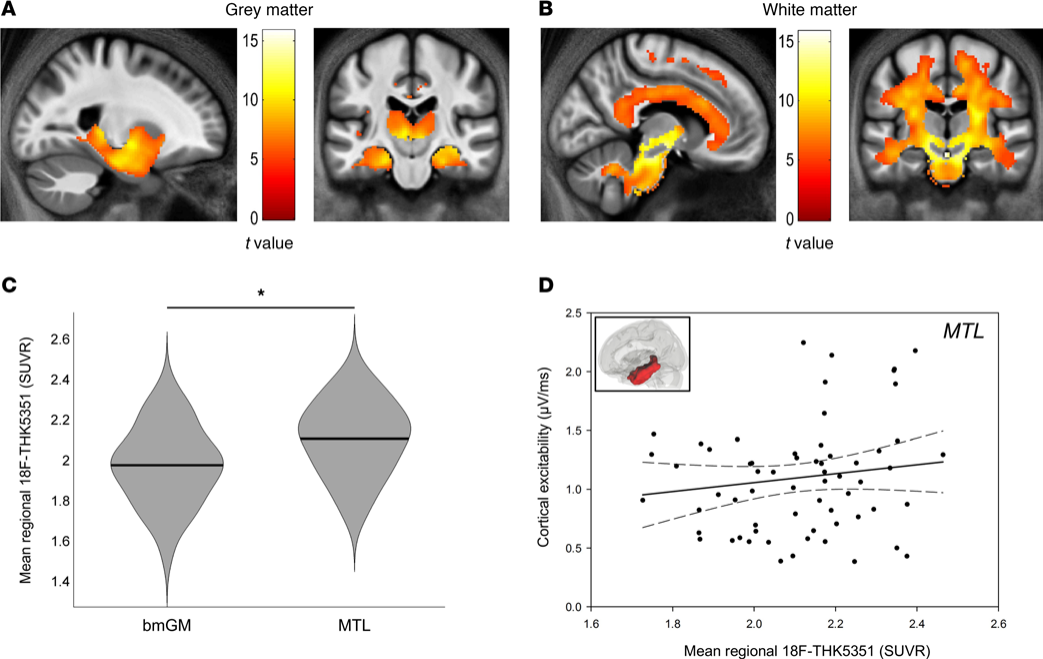
^[18F]^THK5351 regional correlates of brainstem ^[18F]^THK5351 uptake and association between cortical excitability and mean MTL ^[18F]^THK5351 SUVR. (**A**) VBQ analyses revealed that mean ^[18F]^THK5351 SUVR in the brainstem monoaminergic gray matter (bmGM) region was positively correlated to gray matter ^[18F]^THK5351 PET signal within most of the medial temporal lobe (MTL, *P_FWE-corrected_* < 0.001). (**B**) VBQ analyses further showed that mean ^[18F]^THK5351 SUVR in the bmGM region was positively associated with ^[18F]^THK5351 SUVR in the white matter tracts connecting the brainstem to the MTL, but also in widespread projections toward the whole cortex (*P_FWE-corrected_* < 0.001). (**C**) Paired *t* test analysis showed that mean ^[18F]^THK5351 SUVR in the bmGM was significantly lower than in the MTL (*t* = 10.14, *P* < 0.0001). (**D**) Average ^[18F]^THK5351 SUVR in the MTL was, however, not significantly associated with cortical excitability values (Pearson’s correlation: *r* = 0.14, *P* = 0.25; GLMM: F_1,57_ = 1.89, *P* = 0.17). Simple regression is displayed, and full GLMM outputs are reported in Table 4. Dotted lines represent 95% CI for this simple regression.

Our next step was to further address the regional specificity of the association between ^[18F]^THK5351 PET signal and cortical excitability. To do so, we first performed voxel-based quantification (VBQ) analyses on standardized ^[18F]^THK5351 SUVR maps to reveal brain regions for which ^[18F]^THK5351 radiotracer uptake correlated with bmGM uptake. In line with our expectation based on the literature, we found a highly significant correlation with ^[18F]^THK5351 values within most of the medial temporal lobe (MTL; *P* value corrected for family-wise error [*P_FWE-corrected_*] < 0.001; Figure 3A and Table 3). Interestingly, VBQ outputs in the white matter comprised tracts connecting the brainstem to the MTL, as well as widespread projections toward the whole cortex (*P_FWE-corrected_* < 0.001; Figure 3B).

Therefore, we finally extracted mean ^[18F]^THK5351 SUVR over the MTL to test whether the positive association between cortical excitability and ^[18F]^THK5351 PET signal was dependent on the affected region. Paired 1-tailed *t* test analysis showed that mean ^[18F]^THK5351 SUVR was significantly higher in the MTL compared with the bmGM region (*t* = 10.14, *P* < 0.0001; Figure 3C). However, we found no significant relationship between cortical excitability and mean ^[18F]^THK5351 SUVR in the MTL (Pearson’s correlation: *r* = 0.14, *P* = 0.25; GLMM: F_1,57_ = 1.89, *P* = 0.17; Figure 3D and Table 4). Importantly, the correlation value between mean bmGM ^[18F]^THK5351 SUVR and cortical excitability was significantly higher than the correlation value between mean MTL ^[18F]^THK5351 SUVR and cortical excitability (Fisher’s *z* = 2.20, *P* = 0.01).

## Discussion

With this cross-sectional study, we show that ^[18F]^THK5351 uptake in a compartment of the brainstem including monoaminergic neurons is associated with increased cortical excitability in cognitively normal individuals aged 50–69 years. We interpret this result with respect to tau NFT accumulation and neuroinflammation. By contrast, cortical excitability levels are not significantly linked to the presence of Aβ senile plaques measured in their typical earliest aggregation sites. We further find that the brainstem ^[18F]^THK5351 PET signal colocalizes with its uptake in the MTL, in line with human postmortem studies on tau NFT progression (3). However, the association between cortical excitability and ^[18F]^THK5351 uptake in the latter region is not significant. Altogether, these findings support that increased cortical excitability is specifically related to the presence of tau aggregates and neuroinflammation, potentially in a region-specific manner, with the most evident association observed among brainstem monoaminergic nuclei.

^[18F]^THK5351 is a first-generation PET marker of tau NFT that was reported to bind substantially to MAO-B associated with neuroinflammation in addition to binding to tau NFT (31). Antemortem PET studies of ^[18F]^THK5351 SUVR and postmortem neuropathologic studies in progressive supranuclear palsy (32) and AD (29), including ex vivo autoradiography with the selective reversible MAO-B inhibitor lazabemide (32), reported that ^[18F]^THK5351 binding originates, to a large extent (approximately 50%), in reactive astrocytes that express increased levels of MAO-B (31). This unintended binding is, however, most pronounced over the basal ganglia and is also found, although to a lesser extent, in second-generation tau radiotracers (30). Neuroinflammation in astrocytes and microglial dysfunction have been proposed to result from tau-mediated neurodegeneration of LC neurons, potentially through norepinephrine depletion (20, 33, 34). In the rest of the discussion, we therefore interpret brainstem ^[18F]^THK5351 as a marker of tau NFT accumulation and of (potentially tau-induced) neuroinflammation, bearing in mind uncertainties and nonspecificity of the binding. Future studies should employ radiotracers that are more specific to tau protein to further disentangle the respective roles of tau NFT, neuroinflammation, and their interaction in the relationship with increased cortical excitability. Such radiotracers may be available (35, 36) or are still under development (30, 37).

Increased cortical excitability was previously reported in MCI and AD patients — i.e., in the presence of cognitive symptoms (10, 11, 13). Our results show that increased cortical excitability can also be observed in healthy individuals, potentially during preclinical phases of AD — i.e., over the asymptomatic period during which AD-related pathophysiological processes evolve and may lead to subsequent symptomatic AD in some individuals. A rationale often proposed to account for the increased cortical excitability in MCI and AD patients in the first stages of disease postulates that it represents a compensatory mechanism to counteract synaptic dysfunction and tau NFT accumulation within neuronal networks (9). A computational model of neuronal dynamics in AD further reported that the most efficient intervention to counter AD-related network dysfunction and enhance its functional survivability was a selective increase in excitability of excitatory neurons (38). This compensatory phenomenon may, in turn, favor pathological progression as neuronal hyperactivity increases Aβ and tau release (14, 15). Within this scenario, a vicious circle would therefore take place up to a point where Aβ, tau, and neuroinflammation burden and neurodegeneration would be too important to sustain increased excitability (24, 39, 40). This would imply an inverted U-shaped relationship between cortical excitability and network integrity over AD-related pathophysiological processes, with a progressive increase in cortical excitability compensating tau-driven synaptic dysfunction early in the process, followed by decreased excitability in more advanced AD neuropathological stages.

The fact that cortical excitability was associated with tau/neuroinflammation PET measures in a brainstem region including monoaminergic neurons is compatible with the assumption that the association may arise, at least in part, from the LC or from nearby monoaminergic nuclei (e.g., raphe) that are part of the ascending reticular activating system. Tau NFT/neuroinflammation burden in these nuclei could alter their functioning and their impact on distant cortical neurons (e.g., in the prefrontal cortex where we assessed cortical excitability). Higher-resolution PET and MRI data geared toward isolating brainstem nuclei — such as methods based on the neuromelanin-dependent signal of the LC (41) — are, however, required to truly test this assumption.

Previous animal studies reported that cortical excitability increased as a function of tau burden in the hippocampal region or reported reduced cortical excitability following genetic manipulation, reducing overall endogenous tau levels (24, 25, 42, 43). However, those studies did not specifically assess tau presence in the brainstem, even if the literature would suggest that tau accumulation within the LC/brainstem and hippocampal structure should be at least partially concomitant (4). Cortical excitability across the different steps of the stereotypical outward progression of tau NFTs will have to be monitored to resolve this issue. We nevertheless provide original in vivo functional correlates of tau-related PET measures in the brainstem in healthy individuals, with tau spread limited to its earliest stages.

The absence of a significant link between medial prefrontal cortex excitability and Aβ-PET marker uptake in the medial prefrontal cortex and inferior temporal lobe is somewhat unexpected, given the previous links reported in rodents models (27, 44). However, Aβ oligomers and Aβ senile plaques have also been repeatedly associated with lower synaptic spine density, reduced long-term potentiation, and increased long-term depression, as well as reduced neuronal excitability (45, 46). In addition, hyperexcitability was detected in young AD mutant mice, suggesting that increased neuronal excitability was independent of Aβ plaque formation (44). Likewise, efficient Aβ immunotherapy in mice failed to restore normal neuronal function (44). The relationship between AD pathophysiology and neuronal function is, therefore, complex. Overall, our results suggest that the link between frontal cortical excitability and brainstem tau NFT/neuroinflammation burden is more evident than a putative link with medial prefrontal and inferior temporal cortex Aβ senile plaque burden.

Given the cross-sectional nature of our study, we cannot infer about causality, and mechanisms underlying our findings remain elusive. In a first scenario, we propose that the progressive accumulation of misfolded tau protein in the brainstem, particularly over the LC and dorsal raphe nuclei (3), trigger increased cortical excitability as a compensatory mean to face tau NFT accumulation and its associated neuroinflammation. Whole-cell patch-clamp recordings in a mouse model of progressive tauopathy found that frontal cortical neurons exhibit electrophysiological alterations, including a more depolarized resting membrane potential (47), which would result in increased cortical excitability. In addition, altered excitation/inhibition balance and subsequent hyperexcitability may be a consequence of tau accumulation through tau-dependent inhibitory interneurons depletion (48). Although these observations lend support to our first scenario, they were never focused on brainstem tau burden. One cannot exclude, therefore, a second scenario, where increased cortical excitability — which could constitute an endogenous response to environmental factors such as anxiety or lack of sleep — would favor tau production, release, and aggregation, as well as neuroinflammation in the brainstem.

Aside from ^[18F]^THK5351 unspecific binding to MAO, our experiment bears additional limitations. First, we measured cortical excitability exclusively over the frontal cortex. While this area allows for minimally artifacted data and covers the area of earliest Aβ deposit, probing cortical excitability over different parts of the cortex may reveal a broader relationship between early tau/neuroinflammation and alterations of neuronal function. Another limitation of our study is that it only included healthy individuals with relatively limited tau NFT spread. Individuals with more advanced tau pathology, MCI, and early AD patients would have provided further characterization of the link between cortical excitability, AD pathophysiological progression, and especially tau pathology. However, individuals included in our study sample were thoroughly screened for their health and cognitive status, reducing the probability of biases arising from health conditions or AD-related comorbidities. In addition, all individuals’ sleep-wake history was carefully controlled prior to TMS-EEG data acquisition, and cortical excitability measures were acquired in the morning under strictly controlled conditions, after a fixed duration of wakefulness to account for the known changes in cortical excitability with prior wakefulness duration and time of day (49–52). Furthermore, TMS-EEG allowed us to assess cortical excitability directly and reliably, while bypassing any potential age-related sensory biases. Finally, we used state-of-the-art brainstem segmentation methods based on quantitative multiparametric imaging to automatically and systematically isolate brainstem monoaminergic tissue.

In conclusion, the present results point to brainstem tau NFT and neuroinflammation as correlates of the increased excitability observed in the earliest stages of AD neuropathology. These findings bring insights into the interplay between cortical function and the first signs of tau aggregation and its associated neuroinflammation in the brainstem. Furthermore, they suggest that cortical excitability could constitute a specific biomarker of early misfolded tau protein aggregates and neuroinflammation in the brainstem and, potentially, of increased risk for AD in cognitively normal healthy individuals.

## Methods

### Experimental design.

Healthy older individuals aged 50–70 years were enrolled in a multimodal cross-sectional study investigating the relationships between AD neuropathology, wake-dependent cortical excitability dynamics, and cognitive aging. In that context, we measured cortical excitability over the frontal cortex using TMS-EEG, and we performed whole-brain tau/neuroinflammation-PET and Aβ-PET imaging. All participants also underwent quantitative multiparametric MRI acquisitions for subsequent brainstem segmentation. All participants were recruited via local advertisements.

### Participants.

One hundred and one healthy older individuals (mean age = 59.4 ± 5.3 years; 68 women and 33 men) took part in this research. Exclusion criteria for the study were: clinical symptoms of cognitive impairment (dementia rating scale < 130; mini mental state examination < 27; ref. 53); BMI ≤ 18 and ≥ 29; recent psychiatric history or severe brain trauma; addiction or chronic medication affecting the CNS; smoking, excessive alcohol (>14 units/week), or caffeine (>5 cups/day) consumption; shift work in the past 6 months; transmeridian travel in the past 2 months; high levels of anxiety, as measured by the 21-item self-rated Beck Anxiety Inventory (BAI ≥ 17) (54); high levels of depression, as assessed by the 21-item self-rated Beck Depression Inventory (BDI ≥ 17) (55). Participants with sleep apnea (apnea-hypopnea index ≥ 15/hour) were excluded based on an in-lab screening night of standard polysomnography.

A subsample of 65 participants who had data for both tau/neuroinflammation-PET and Aβ-PET assessments was considered for the present paper. One participant was further excluded from the study sample because of extreme outlier values on whole-brain ^[18F]^THK5351 PET assessment (>5 SDs from the mean). Demographic characteristics of the final sample (*n* = 64) are described in Table 1.

### Experimental protocol.

For 7 days prior to TMS-EEG protocol, participants followed a regular sleep-wake schedule (±30 minutes), in agreement with their preferred bed and wake-up times. Compliance was verified using sleep diaries and wrist actigraphy (Actiwatch, Cambridge Neurotechnology). Aside from the fixed sleep-wake schedule, participants were also instructed to abstain from unusual physical exercise, as well as caffeine and alcohol consumption, for the last 3 days of fixed sleep-wake schedule. The day before the experiment, participants arrived to the laboratory 8 hours before their habitual bedtime and were kept in dim light (< 5 lux) for 6.5 hours preceding bedtime. Participants then slept a full night of sleep, recorded with EEG.

TMS-EEG assessments were performed on the next day in the context of a 20-hour protocol of wakefulness extension in strictly controlled constant routine conditions — i.e., in-bed semirecumbent position (except for scheduled bathroom opportunities), dim light < 5 lux, temperature ~19°C, regular isocaloric food intake, no time-of-day information, and sound-proofed rooms. The protocol schedule was adapted to individual sleep-wake time and lasted up to the theoretical midsleep time (e.g., for an individual waking up at approximately 07:00 am, the protocol ended at approximately 03:00 am the following night). Cortical excitability over the frontal cortex was measured 5 times throughout the protocol. For the present paper, we only considered the first assessment of cortical excitability recorded 3 hours after wake-up time (±0.21 hours) to avoid any wake-dependent modulation of cortical excitability (51, 52). Other considerations related to repeated TMS-EEG sessions are reported elsewhere (51).

### TMS-EEG signal acquisition and processing.

Optimal stimulation parameters (i.e., location, orientation, and intensity) were determined during a separate TMS-EEG session carried out prior to the experimental protocol, and they allowed for EEG recordings free of muscular and magnetic artifacts. As in previous experiments (49, 50, 52), the target location was in the superior frontal gyrus. For all TMS-EEG recordings, pulses were generated by a Focal Bipulse 8-Coil (Nexstim). Interstimulus intervals were randomized between 1900 and 2200 ms. TMS-evoked responses were recorded with a 60-channel TMS-compatible EEG amplifier (Eximia), equipped with a proprietary sample-and-hold circuit, which provides TMS artifact–free data from 5 ms after stimulation (56). Electrooculogram (EOG) was recorded with 2 additional bipolar electrodes. EEG signal was band-pass filtered between 0.1 and 500 Hz and sampled at 1450 Hz. Before each recording session, electrode impedance was set below 5 kΩ. Each TMS-EEG session included approximately 250 trials. Auditory EEG potentials evoked by the TMS clicks and bone conductance were minimized by diffusing a continuous white noise through earphones and applying a thin foam layer between the EEG cap and the TMS coil.

TMS-EEG data were preprocessed as previously described (50, 52) in Statistical Parametric Mapping 12 (SPM12, http://www.fil.ion.ucl.ac.uk/spm/) implemented in MATLAB2013a (The Mathworks Inc.). In brief, TMS-EEG data underwent semiautomatic artifact rejection, low-pass filtering at 80 Hz, downsampling to 1000 Hz, high-pass filtering at 1 Hz, splitting into epochs spanning –101 and 300 ms around TMS pulses, baseline correcting (from –101 to –1 ms pre-TMS), and robust averaging. Cortical excitability was defined as the slope at the inflexion point of the first component (0–35 ms) of the TEP measured on the artifact-free electrode closest to the target stimulation location. Median position of the closest artifact-free recording electrode was (*x*, –34; *y*, –3.7; *z*, 85.9, mm, Montreal Neurological Institute [MNI] space).

### Quantitative multiparametric MRI acquisition and processing.

All MRI acquisitions were performed on a 3-T scanner (MAGNETOM Prisma, Siemens). Structural and quantitative maps of T1, T2*, proton density (PD) and magnetization transfer (MT) with 1 mm isotropic resolution were computed based on a multiparameter protocol including 3D multiecho fast low angle shot (FLASH) sequence (57). Three colocalized 3D multi-echo FLASH data sets were acquired with predominantly PD weighting (PDw: repetition time/flip angle [TR/FA] = 23.7 ms/6°), T1 weighting (T1w: 136166 = 18.7 ms/20°), and MT weighting (MTw: TR/FA = 23.7 ms/6°; excitation preceded by an off-resonance Gaussian MT pulse of 5 ms duration, 220° nominal flip angle, 2 kHz frequency offset) in a total acquisition time of approximately 19 minutes, with a voxel size of 1 mm^3^ isotropic. Two calibration sequences were acquired to correct for inhomogeneities in the radio frequency transmit field.

Quantitative multiparametric maps (MT, PD, R1, R2*) were generated with the hMRI toolbox (58) (http://hmri.info) implemented in MATLAB2013a. First, MT and PD maps were segmented into gray, white, and CSF tissue class maps using Unified Segmentation (US) within SPM12 (59). Whole-brain segmentation outputs were diffeomorphically registered to a study-specific template, compatible with the MNI space, created using Shoot toolbox in SPM12 (60) in order to generate deformation fields that were used to warp MT and PD maps into the study-specific average space. Brainstem segmentation was then performed using US with brainstem subregion tissue probability maps that were generated according to previously described methods based on a modified multivariate mixture of Gaussians (61). Out of the 4 brainstem tissue classes produced, we considered only tissue class 1, which encompassed bmGM, including LC and raphe nuclei. bmGM tissue was then warped back to individual space, using inverse deformation fields. Finally, bmGM tissue was binarized and applied as a mask on coregistered PET ^[18F]^THK5351 for signal extraction, as described below.

### PET acquisition and preprocessing.

Tau/neuroinflammation and Aβ-PET imaging were performed on an ECAT EXACT+ HR scanner (Siemens), with a 2 mm isotropic resolution. Tau/neuroinflammation PET imaging was performed with radiotracer ^[18F]^THK5351 for all subjects. Aβ-PET imaging was achieved with radiotracer ^[18F]^Flutemetamol for 61 subjects and with ^[18F]^Florbetapir for 3 subjects. For all PET imaging, participants received a single dose of the respective radiotracer in an antecubital vein (target dose approximately 185 MBq). For tau/neuroinflammation-PET, a 10-minute transmission scan was first acquired, and dynamic image acquisitions started immediately after injection, consisting of 32 frames with increasing time duration (total time spent in scanner, approximately 100 minutes). For Aβ-PET, image acquisitions started 85 minutes after injection, and 4 frames of 5 minutes were obtained. All PET images were reconstructed using a filtered back-projection algorithm, including corrections for measured attenuation, dead time, random events, and scatter using standard software (ECAT 7.1, Siemens/CTI). For each individual, an average PET image was created using all frames for Aβ-PET, and the 4 frames corresponded to the time window between 40 and 60 minutes for tau/neuroinflammation-PET (62). Averaged PET images were manually reoriented and automatically coregistered to the structural MT map.

SUVR was calculated using the cerebellum gray matter as the reference region for tau/neuroinflammation-PET (32) and the whole cerebellum for Aβ-PET (63). Given that we used 2 different radiotracers for Aβ-PET imaging, Aβ SUVR values were further scaled to centiloid units (63–65). Subject-space bmGM mask was applied on ^[18F]^THK5351 SUVR maps to retrieve mean tau/neuroinflammation burden. An ROI comprising the earliest Aβ accumulation sites (stage 1 ROI) (7) was built based on bilateral regions from the automated anatomical labelling 2 (AAL2) atlas (66), including superior medial frontal, fusiform, and inferior temporal cortices, and it was applied on the standardized Aβ maps.

Mean ^[18F]^THK5351 SUVR was 1.43 ± 0.11 over the whole brain, and 1.97 ± 0.20 in the bmGM. Mean whole-brain Aβ centiloid value was 2.47 ± 9.92, and –5.31 ± 8.20 in the stage 1 ROI. The negative centiloid values in these earliest Aβ accumulation sites were expected, as our sample comprises only healthy participants in late middle age who were thoroughly screened for absence of many comorbidity factors.

While first-generation tau protein radiotracers, including ^[18F]^THK5351, have been widely studied and proved to have high affinity and selectivity in vitro (31), criticism was raised about their off-binding to MAO-B in vivo, particularly over the basal ganglia (67). However, as investigated in a recent comparative analysis of first- and second-generation radiotracers, this off-target binding may be common to all first-generation radiotracers and, to a lesser extent, also to second-generation radiotracers (30). We therefore consider that ^[18F]^THK5351 uptake arises from about equal proportion of binding to both tau NFT and MAO-B (31), and it constitutes a marker of both tau and neuroinflammation burden.

### Tau colocalization analysis.

Spatial processing of PET images for VBQ analyses was achieved with the standard pipeline module in hMRI toolbox. Whole-brain ^[18F]^THK5351 SUVR maps were diffeomorphically registered to the study-specific template using individual deformation fields, warped into the MNI space, and tissue-specific smoothing (full width at half-maximum of 6 mm isotropic, separately for gray and white matter) was applied. VBQ analyses were carried out on these images in SPM12 in order to highlight brain regions that correlate with tau/neuroinflammation burden in the bmGM tissue. Statistical modeling consisted of a multiple linear regression model including 4 regressors (i.e., demographic variables of age, sex, education, and bmGM mean ^[18F]^THK5351 SUVR). One-tailed *t* tests were used to identify voxels displaying increased ^[18F]^THK5351 SUVR associated with higher values in the bmGM compartment. Statistical threshold was set at *P* < 0.05 after FWE correction for multiple comparisons (*P_FWE-corrected_*) voxel-wise for the whole-brain. Gray matter and white matter tissue classes were analyzed separately using explicit masks defining voxels belonging to each tissue class. These masks were generated using the across-subjects average of smoothed Jacobian-modulated tissue probability maps in MNI space, with a minimum probability threshold of 20%, and voxels were assigned to the tissue class for which the probability was maximal. For both VBQ analyses, bmGM area was further masked to avoid redundancy.

### Statistics.

Statistical analyses were performed using Pearson’s correlations and GLMMs in SAS 9.4 (SAS Institute). Pearson’s correlations were used as an assessment of potential association before computing GLMM, which accounted for possible biases and covariate influences. Dependent variable distribution was first determined using “allfitdist” function in MATLAB2013a, and GLMMs were adjusted accordingly. TMS-EEG stimulation parameters of applied electric field and distance between hotspot and recording electrode were included as covariates in GLMM statistical models with cortical excitability measures as dependent variables. All GLMMs were adjusted for demographic variables of age, sex, and education. Subject (intercept) was included as a random factor. Degrees of freedom were estimated using Kenward-Roger’s correction. Statistical significance was set at *P* < 0.05. Semi-partial *R²* (*R*²**_β*_**) values were computed to estimate the effect sizes of significant fixed effects in all GLMMs (68). A 1-tailed paired *t* test was used to compare mean ^[18F]^THK5351 SUVR in the bmGM and in the MTL.

### Study approval.

This study was approved by the Ethics Committee of the Faculty of Medicine at the ULiège, Belgium. Participants gave their written informed consent prior to inclusion in the study and received financial compensation.

## Author contributions

ES, CB, FC, CP, PM, and GV designed the study. MVE, DC, JN, GB, VM, CS, DM, PC, NV, MG, AL, ES, CL, CB, FC, CP, PM, MAB, EB, and GV helped in data acquisition, analysis, and interpretation. ES, AL, PM, and CP provided administrative, technical, or material support. MVE and GV wrote the paper. MVE, DC, JN, GB, VM, CS, DM, PC, NV, MG, AL, ES, CL, CB, FC, CP, PM, MAB, EB, and GV contributed to manuscript revision.

## Supplementary Material

Supplemental data

## Figures and Tables

**Table 1:**
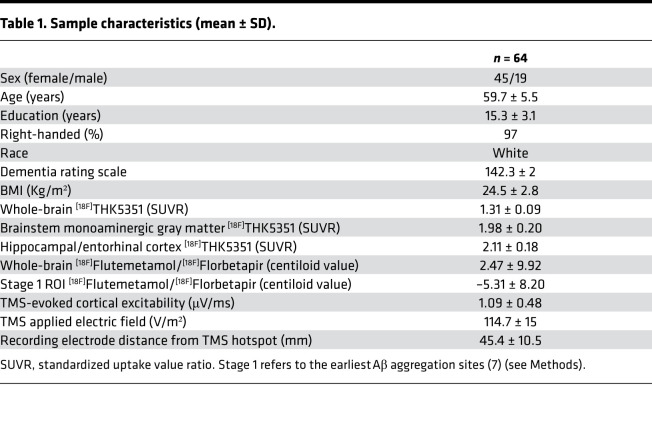
Sample characteristics (mean ± SD).

**Table 2:**
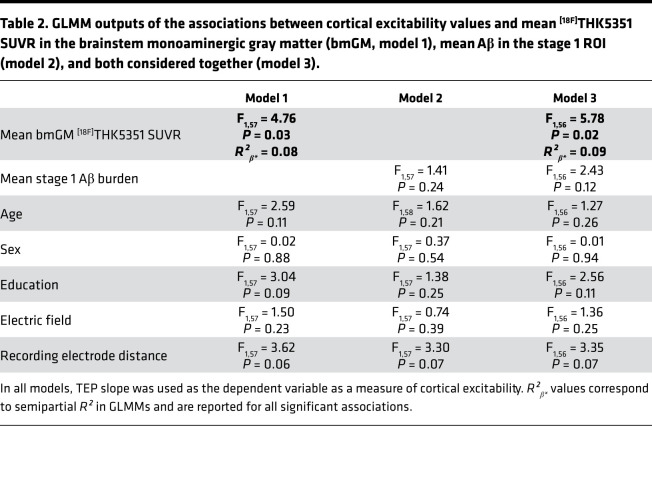
GLMM outputs of the associations between cortical excitability values and mean ^[18F]^THK5351 SUVR in the brainstem monoaminergic gray matter (bmGM, model 1), mean Aβ in the stage 1 ROI (model 2), and both considered together (model 3).

**Table 3:**
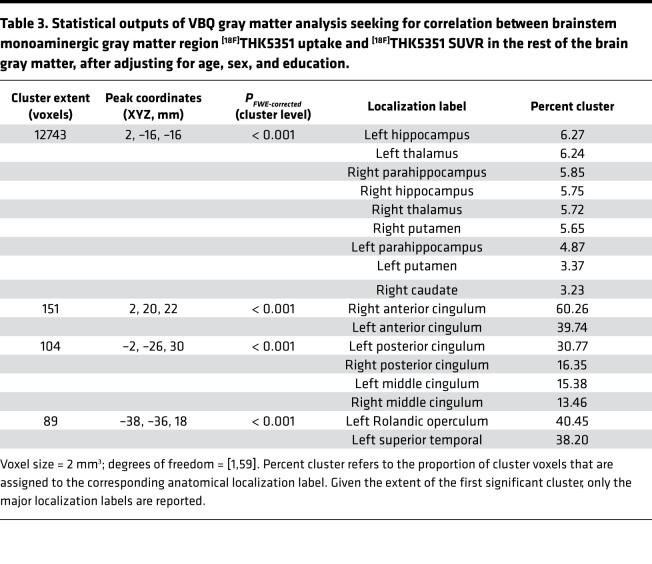
Statistical outputs of VBQ gray matter analysis seeking for correlation between brainstem monoaminergic gray matter region ^[18F]^THK5351 uptake and ^[18F]^THK5351 SUVR in the rest of the brain gray matter, after adjusting for age, sex, and education.

**Table 4:**
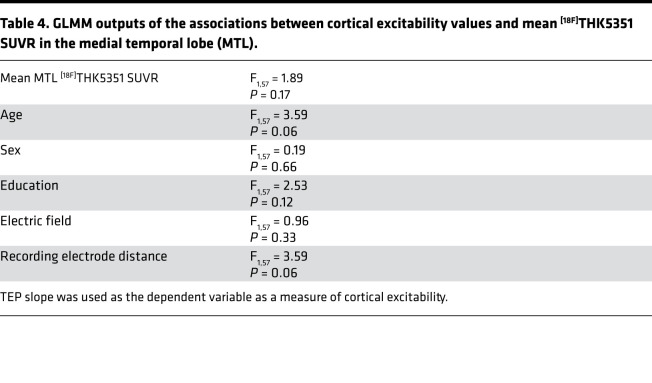
GLMM outputs of the associations between cortical excitability values and mean ^[18F]^THK5351 SUVR in the medial temporal lobe (MTL).

## Version 1 12/08/2020

In-Press Preview

## Version 2 01/25/2021

Electronic publication
